# Evaluation of a new out-of-hospital newborn life support (OH-NLS) course in the UK South West region: a mixed-methods survey study

**DOI:** 10.29045/14784726.2024.12.9.3.44

**Published:** 2024-12-01

**Authors:** Michael Bradfield, Laura Goodwin, Sarah Bates, Robert Tinnion, Sally Hedge, Dawn Kerslake, John Madar, Lucy Murcott, Wendy Tyler, James Yates, Anna Powell, Louise Hall

**Affiliations:** Resuscitation Council UK; Bournemouth University ORCID iD: https://orcid.org/0000-0003-1131-3638; University of the West of England ORCID iD: https://orcid.org/0000-0002-9118-4620; Great Western Hospitals NHS Foundation Trust ORCID iD: https://orcid.org/0000-0002-0646-7440; Newcastle upon Tyne Hospitals NHS Foundation Trust; Health Innovation South West; South East Coast Ambulance Service NHS Foundation Trust; University Hospitals Plymouth NHS Foundation Trust ORCID iD: https://orcid.org/0009-0000-9048-3277; Black Country Local Maternity and Neonatal Transformation System; Shrewsbury and Telford Hospital NHS Trust ORCID iD: https://orcid.org/0009-0002-5929-2012; University Hospitals Bristol and Weston NHS Foundation Trust ORCID iD: https://orcid.org/0000-0002-2520-0602; Health Innovation South West; Health Innovation South West

**Keywords:** education, emergency medical services, infant, newborn, resuscitation

## Abstract

**Introduction:**

Unplanned out-of-hospital births (UOHBs) are associated with poorer outcomes for babies, especially those born prematurely. The current Newborn Life Support (NLS) course offered by Resuscitation Council UK (RCUK) is not designed to address the challenges associated with birth out of hospital. A new course was developed to address these challenges. This study aimed to evaluate the impact of this course on attendees’ knowledge and confidence in supporting transition, resuscitation, stabilisation and onward transfer of newborns in an out-of-hospital setting.

**Methods:**

A convergent mixed-methods approach was used consisting of pre-, post- and follow-up surveys and a post-course multiple-choice questionnaire (MCQ). The surveys asked participants to rate their confidence, on a five-point Likert scale (from ‘Underconfident/fearful’ to ‘Very confident’) across seven domains of NLS, as well as making an individual assessment of provider confidence before and after the course. Free-text comments were collected and analysed using thematic analysis.

**Results:**

Attendees comprised multidisciplinary staff from the South West of England. The pre-course survey was completed by 32 of the 33 participants, the post-course survey by 31 and the MCQ by all 33. A total of 18 participants completed the follow-up survey. Analysis showed a significant, positive change in confidence across NLS domains between the pre- and post-course surveys (p <0.0001).The follow-up survey data showed self-reported increases in knowledge and largely sustained confidence. The qualitative analysis revealed themes relating to the participants’ feelings about managing babies born out of hospital.

**Conclusion:**

The proof-of-concept OH-NLS course appears to address the learning needs of the target professional group, and the results suggest improved knowledge and confidence in the immediate management of babies born out of hospital. Further evaluation is required to determine whether such training has a long-term impact and translates into improved outcomes across a larger group of participants.

## Introduction

Birth outside the hospital environment may be defined as either planned or unplanned. For multiparous women deemed to be at low risk of complications, birthing in the home environment with appropriate support has been shown to be as safe as birth in hospital (Birthplace in England Collaborative Group, 2011). However, unplanned out-of-hospital birth (UOHB) is associated with poorer outcomes for babies (Javaudin et al., 2019; Ovaskainen et al., 2015; Snowden et al., 2015). For preterm babies, mortality has been reported to be more than twice as high for UOHB than in-hospital matched controls (Jones et al., 2011). Severe perinatal brain injury has been associated with an early post-natal transfer for extremely preterm babies (Helenius et al., 2019). Reduction in neonatal mortality and brain injury is a national priority in England (National Health Service, 2019).

Rates of UOHB attended by emergency ambulance services in the UK are around 4000 a year (Goodwin et al., 2022; McClelland et al., 2019; Office for National Statistics, 2019). The rate of such births may be increasing internationally (Australian Institute of Health and Welfare, 2023; Ovaskainen et al., 2015). Most UOHBs in an Australian study were uncomplicated, precipitous term births (McLelland et al., 2018). However, 11% of babies were born before 36 weeks’ gestation and of these, just over a third were born between 24 and 32 weeks’ gestation. Research shows that mothers giving birth in the out-of-hospital setting are more likely to be multiparous, have increased rates of perinatal complication and their labour is more likely to occur out of hours (Bhoopalam & Watkinson, 1991; Goodwin et al., 2023; Loughney et al., 2006). There may be higher than baseline rates of preterm birth and perinatal complications to contend with outside of hospital (McLelland et al., 2018).The most common neonatal morbidity following UOHB is hypothermia, defined as a temperature of <36.5°C (World Health Organization, 1997). Neonatal hypothermia can lead to hypoxia, hypoglycaemia and acidosis. For premature (<37 weeks) or low birthweight (<2500 g) infants, mortality has been shown to increase by 28% per 1°C decrease in admission temperature below 36.5°C (Laptook et al., 2007). Despite the known risk of hypothermia following UOHB, neonatal temperature has been found to be poorly recorded by ambulance staff, ranging from just 2% to 10% of cases in published audits. When neonatal temperatures were recorded out of hospital, the vast majority were below <36.5°C (Flanagan et al., 2017; Goodwin et al., 2022; McClelland et al., 2019). A large proportion of babies remained hypothermic on arrival at hospital (Goodwin et al., 2023; Javaudin et al., 2020; Ovaskainen et al., 2015). Even modest improvements to rates of hypothermia could be associated with improved outcomes for these babies, yet ambulance providers are reported to feel unprepared to deal with newborn infants (Goodwin et al., 2022; Hill et al., 2023; Madar et al., 2017; Persson et al., 2019; Vagle et al., 2019).

Resuscitation Council UK (RCUK) offers a well-established NLS course for healthcare professionals involved in the delivery and care of the newborn (RCUK, 2023a). While some out-of-hospital practitioners in the UK do attend this course, it is not designed to address the different environmental, logistic, teamworking, transport and equipment challenges encountered outside a hospital setting.

## Methods

A working group was established in May 2022 to adapt the core NLS course material and principles to an out-of-hospital setting. This consisted of an experienced multidisciplinary faculty representing the major professional groups involved in attending community births and resuscitation. Two out-of-hospital NLS (OH-NLS) courses were delivered in November 2022 (Supplementary 1). The evaluation was funded by Health Innovation South West and New Life Special Care Babies (2023).

### Participants

Participants in this study were attendees on two OH-NLS courses, working in the South West of England, covering rural and urban areas with a range of population densities.

### Technical information

The aim of the study was to assess the impact of the training on participants’ levels of knowledge and confidence in supporting transition, stabilisation, resuscitation and onward transfer of newborns outside of hospital.

For this convergent mixed-methods study, both qualitative and quantitative data were gathered through surveys and questionnaires framed around three of the four levels of the Kirkpatrick (1996) model of training evaluation (reaction, learning, behaviour and results).

A total of 33 participants attended over two courses. Attendees were asked to complete a pre-course, post-course and follow-up survey (hosted on Microsoft (MS) Forms) to evaluate the course (Supplementary 2‒4), and a paper-based post-course multiple-choice questionnaire (MCQ) to assess their learning. Participants were asked to put their candidate numbers on the surveys to match pre-course, post-course and follow-up data but, for anonymity, those performing the analysis were not aware of names or professional identity, and comments are not identifiable to individual participants. Informed consent was obtained at the start of the survey, with participant information about the use of data being displayed on the welcome page.

Each of the surveys asked participants to rate their confidence, on a five-point Likert scale (‘Underconfident/fearful’ to ‘Very confident’), across seven domains of NLS:

1. Airway management for a newborn baby.
2. Cardiac compressions for a newborn baby.
3. Management of a preterm baby at birth.
4. Thermal care for a newborn baby.
5. Transportation of a newborn baby.
6. Assessment of the need for the apparently well baby to be taken into hospital.
7. Communication with the parents about the condition of the baby.

Participants were asked to share their overall feeling of confidence in their ability to deliver optimal resuscitation support to a newborn baby in the community setting. The surveys used free-text questions about prior experience and reaction to the training. The pre-course survey was distributed via email by the course director (October‒November 2022). The post-course survey was distributed via survey link and QR code, and the follow-up survey was distributed eight months after the training, via email (June‒July 2023). The post-course MCQ consisted of 50 true/false questions, with a pass mark of 80%, at the same standard as the existing RCUK NLS course.

### Analysis

For the quantitative analysis, survey responses were exported from MS Forms to an MS Excel spreadsheet and converted into numeric interval-level data (‘Underconfident/fearful’ = 1; ‘Very confident’ = 5), and the sum of pre-course, post-course and follow-up survey responses was calculated for each participant. Differences between participants’ responses at each of the three time points were assessed using a paired Wilcoxon signed-rank test.

Qualitative responses to the free-text survey questions were analysed using thematic analysis (Braun & Clarke, 2006). Free-text responses were reviewed to allow familiarity with the data, with initial codes generated using open coding and an inductive approach. As the dataset was not large, this was done manually by one researcher, with codes being refined and reapplied to the whole dataset using an iterative process to develop themes.

## Results

The pre-course survey was completed by 32 attendees, 31 completed the post-course survey and 18 completed the follow-up survey. Pre- and post-course surveys could be matched for 27 candidates, of which 21 had complete data on both. All 33 attendees completed the graded, post-course MCQ.

### Participant characteristics

Participants’ job titles can be seen in Table 1. The surveys did not record demographic information, to facilitate anonymity within a relatively small group.

**Table 1.** Participant characteristics.

Participant job title	Frequency
Paramedic	15 (45%)
Community Midwife	12 (36%)
Emergency Care Assistant	2 (6%)
Hospital Midwife	1 (3%)
Nurse	1 (3%)
Pre-hospital Emergency Medicine Doctor	1 (3%)
Unknown	1 (3%)
**Frequency previously called on to provide NLS**	
0 times	17 (53%)
1 time	5 (16%)
2 times	5 (16%)
3 times	1 (3%)
6 times	1 (3%)
8 times	1 (3%)
10 times	2 (6%)
**Previous training (within last four years)**	
Less than one day	19 (59%)
1 day	11 (34%)
2 or more days	2 (6%)

Most (94%) participants had received a day or less of previous training in the past four years. More than half (17/32, 53%) had not been called on to provide NLS outside of hospital in the last four years (see Table 1).

Results of the course evaluation are structured within the Kirkpatrick levels of training evaluation (reaction, learning and behaviour).

#### Reaction: perspectives on the course

In the post-course survey, participants reported being ‘Very satisfied’ with the content (30/31, 97%), structure (29/31, 94%) and pace (27/31, 87%) of the course, as well as the venue (31/31, 100%) and materials (28/31, 90%).

#### Learning: knowledge and confidence

Scores from the MCQ ranged between 76% and 100% (mean 87%, SD 6.9). Most participants (29/33, 88%) reached the pass mark. It is not known whether this may have improved as a result of the course alone and to what extent this knowledge was retained in the longer term or translated into practice.

**Figure 1:**
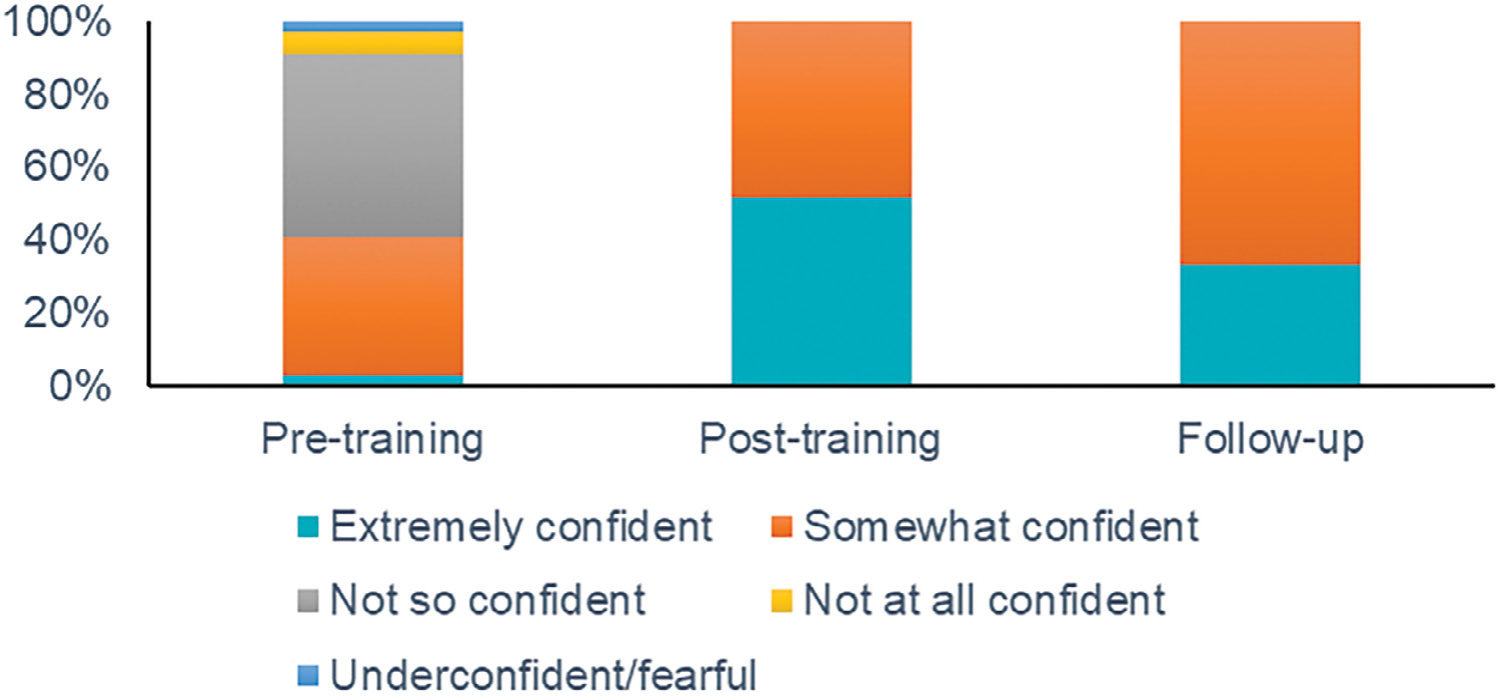
Figure 1. Self-reported ratings of overall confidence over time in the management of a newborn baby outside hospital.

The percentage of participants reporting their overall feeling of confidence in ability to deliver optimal NLS in the community setting as ‘Extremely confident’ increased from 3% (1/32) before the course to 52% (16/31) afterwards. Eight months after the course, 33% (6/18) of participants reported feeling ‘Very confident’, suggesting a fade in confidence but still considerably higher than the pre-course level. All responses are shown in Figure 1.

Table 2 shows a noticeable shift in the participants’ self-reported confidence in all seven domains before and after the course and at follow-up.

**Table 2.** The proportion of participants reporting feeling ‘Very confident’ in their NLS abilities before and after the course.

Aspect of care	Pre-course (n = 32) N (%)	Post-course (n = 31) N (%)	Follow-up (n = 18) N (%)
1. Airway management for a newborn baby	2 (6%)	16 (59%)	10 (56%)
2. Cardiac compressions for a newborn baby	1 (3%)	20 (74%)	13 (72%)
3. Management of a preterm baby at birth	0 (0%)	8 (30%)	7 (39%)
4. Thermal care for a newborn baby	4 (13%)	24 (89%)	17 (94%)
5. Transportation of a newborn baby	1 (3%)	10 (37%)	9 (50%)
6. Assessment of the need for the apparently well baby to be taken into hospital	2 (6%)	15 (56%)	8 (44%)
7. Communication with the parents about the condition of the baby	2 (6%)	15 (56%)	14 (78%)

For five of the seven domains (1, 2, 4, 6 and 7), 100% of participants reported feeling either ‘somewhat’ or ‘very’ confident in their ability immediately after attending the course and at follow-up.

**Figure 2:**
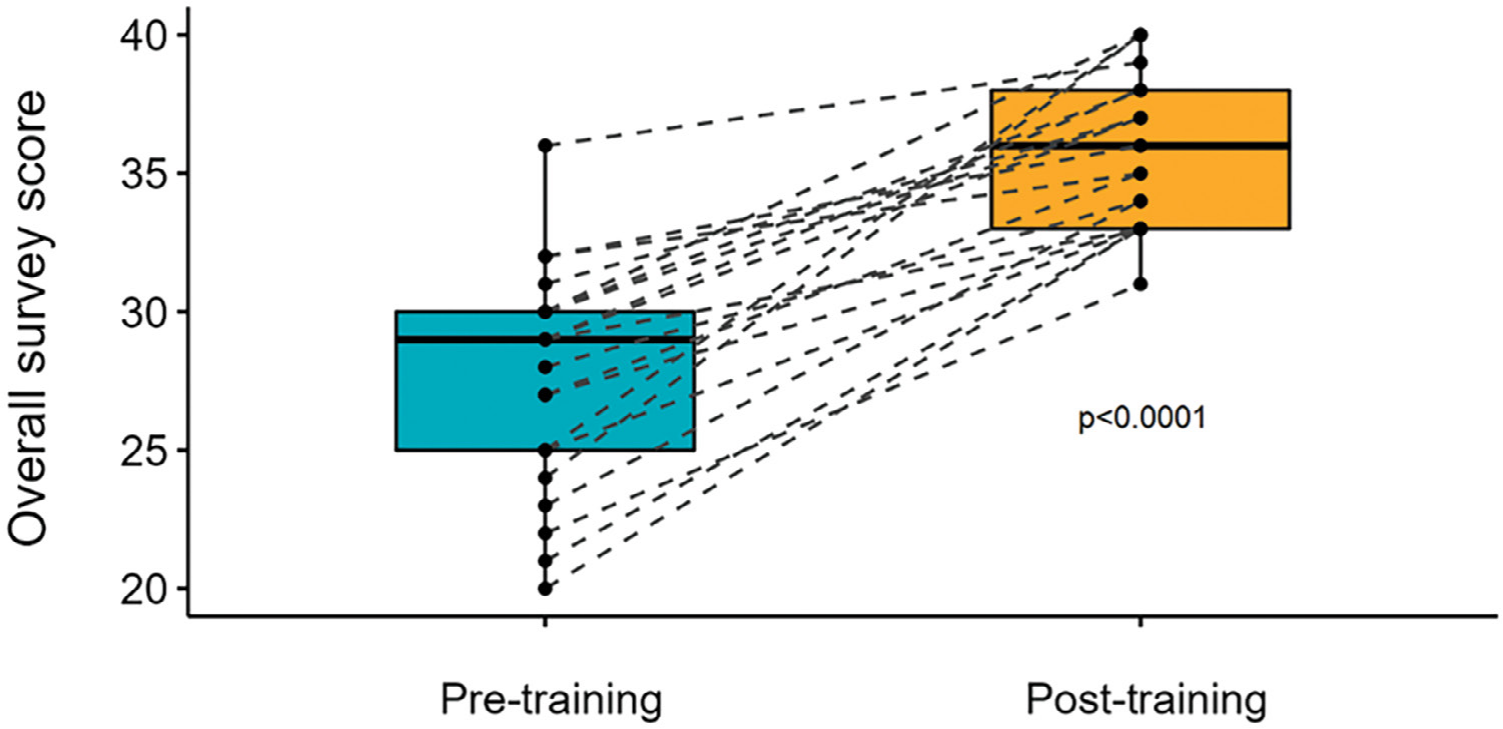
Figure 2. Comparisons of combined domain (average total score across domains), matched pre- and post-course self-reported confidence in managing a newborn baby outside hospital.

The results of the comparison analysis showed a significant difference in the confidence between pre- and post-course surveys (n = 21, Z = -4.014, p <0.0001) (Figure 2).

**Figure 3:**
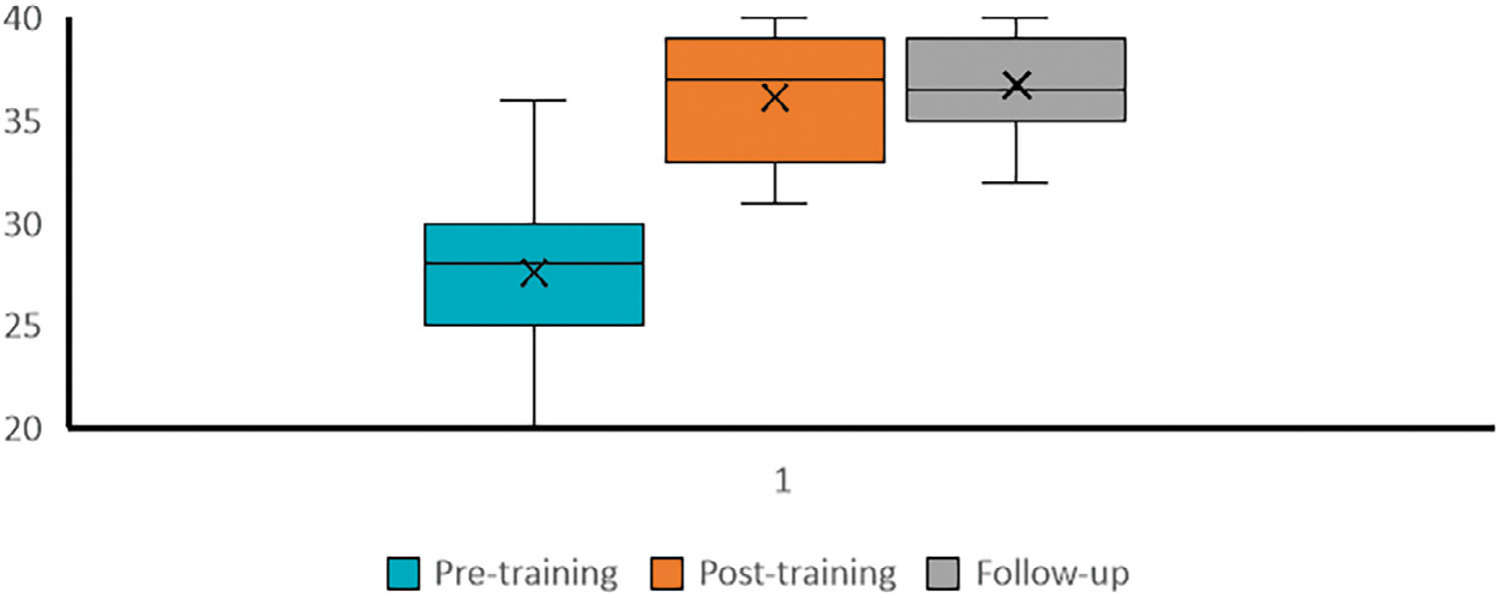
Figure 3. Comparisons of all respondents’ combined domain (average total score across domains) pre-, post- and follow-up self-reported confidence in managing a newborn baby outside of hospital.

Observation of the average scores over time (Figure 3) suggests that confidence was maintained at follow-up for those completing the follow-up survey.

Follow-up survey data showed self-reported increases in knowledge across all the different aspects of care and stabilisation of a newborn baby outside hospital (Table 3).

**Table 3.** The proportion of participants reporting an increase in their knowledge and understanding of aspects of newborn life support after attending the course (at follow-up, n = 18).

Aspect of care	Some increase N (%)	Significant increase N (%)	Total
1. Airway management for a newborn baby	7 (39%)	10 (56%)	17 (94%)
2. Cardiac compressions for a newborn baby	7 (39%)	7 (39%)	14 (78%)
3. Management of a preterm baby at birth	3 (17%)	15 (83%)	18 (100%)
4. Thermal care for a newborn baby	4 (22%)	14 (78%)	18 (100%)
5. Transportation of a newborn baby	5 (28%)	10 (56%)	15 (83%)
6. Assessment of the need for the apparently well baby to be taken into hospital	7 (39%)	9 (52%)	16 (89%)
7. Communication with the parents about the condition of the baby	6 (33%)	7 (36%)	13 (72%)
8. Appropriate assessment of the baby immediately after birth	5 (28%)	11 (61%)	16 (89%)
9. Communication with a multidisciplinary team around the optimal management of the newborn baby	8 (44%)	8 (44%)	16 (89%)
10. Cord management	5 (28%)	9 (50%)	14 (78%)

#### Behaviour: impact of the course as perceived by attendees

Half (9/18) of the participants who responded to the follow-up survey stated that they had experienced the opportunity to implement their learning from the course. In the follow-up survey, eight participants described a scenario where they had implemented learning from the course. When asked the extent to which they felt the course was helpful in how they approached the situation, 38% (3/8) reported that it was ‘critical’, and 63% (5/8) reported it as ‘very helpful’.

### Qualitative findings

Three themes were identified from the free-text responses across five questions (Supplementary 5), providing insight into how participants felt about NLS before and following the training.

#### Theme 1: lack of exposure and training

Prior to the course, participants felt that the infrequent nature of the requirement for NLS led to feelings of fear and anxiety and a lack of confidence.

*As a paramedic, I have received limited training in newborn care. In my third year of university (2021), we had a day training in resuscitation following birth. In my paramedic induction we spent less than one hour focusing on newborn care/resuscitation . . . we do not have regular training in this, which causes underconfidence in practice, whilst I know how to care/stabilise/transport a newborn, it can be daunting due to the lack of training.*(Participant 7)

Even those who were confident pre-course felt the lack of frequent exposure would lead to skill fade, so frequent training should be offered. These thoughts were echoed following the training where participants cited ‘skill fade’ and ‘lack of practice’ as barriers to performance.

#### Theme 2: practical benefits of the training

This theme recognises the difference the course made to the system when some of the participants took the opportunity to apply their learning. The course triggered attendees to run training courses and source new equipment:

*Transwarmers purchased for all homebirth bags. Myself and my colleague have run training sessions for community midwives on thermoregulation and resus in pre-hospital settings.* (Participant 10)

*We have taken to the management team the need for iGels in all homebirth bags and encouraged this into our mandatory NLS training. All midwives are receiving training to use iGels now.* (Participant 33)

*In process of introducing the penguin suction device to homebirth bags.* (Participant 4)

The course also provided attendees with the confidence to talk to parents and to manage events ‘in a calm way’ (Participant 27). Some attendees referenced the application of their learning into real-life situations. One managed a ‘neonatal resus at home birth followed by transfer into hospital’ (Participant 14) and a few noted the benefit of training across providers, one stating that it was ‘really good to build knowledge and found it [the course] extremely useful being based in the community with the paramedics that we are likely to work with’ (Participant 14).

#### Theme 3: systemic barriers to caring for newborns born out of hospital

Participants noted the challenges beyond individual provider training that need to be addressed to support community-based resuscitation outcomes across the system. Several participants cited a lack of appropriate or available equipment as a barrier to effective care.

*The lack of specialist/neonatal kit ambulances carry.* (Participant 2)

*Equipment available in the community setting.* (Participant 21)

Some referenced ambulance delays as a potential problem that causes them anxiety, while others described the difficulties in managing situations with colleagues who have not attended the training.

*Understanding from the wider cohort of paramedics who were questioning the correct procedures that then they agreed with but without my intervention delayed care would have occurred.* (Participant 22)

More and wider-reaching training to ‘all colleagues in flash teams’ was called for to support these issues.

## Discussion

Findings from the pre-course data support other published literature on the experiences of pre-hospital and community practitioners (Goodwin et al., 2022; Heys et al., 2022; Hill et al., 2023) by confirming that there remains an unmet need for access to training on NLS applicable to the out-of-hospital setting. While the frequency of attending scenarios where NLS is needed in the community setting is low, their nature as ‘high acuity, low occurrence’ (HALO) events means that they remain ever-present in the practitioners’ minds as daunting, anxiety-inducing possibilities (Goodwin et al., 2022; Heys et al., 2022; Hill et al., 2023; Persson et al., 2019).

Evaluation of the OH-NLS course found that it improved practitioners’ self-reported knowledge and confidence in all aspects of delivering care to stabilise a newborn baby outside the hospital setting. This increased level of confidence was sustained at eight months after course attendance, although those reporting to be ‘extremely confident’ fell from 52% to 33%. Findings suggested that the course was effective in improving confidence in two key areas of neonatal care delivery ‒ airway management and thermal care ‒ areas in which improved care delivery could offer significant improvements in outcomes for those born outside hospital (Chitty & Wyllie, 2013; Javaudin et al., 2019; Nguyen et al., 2016).

Practitioners reported that the pre-course materials, the teaching on the day and the assessments used at the end of the course were useful, helpful and appropriate. Participants felt that learning was strengthened by the inclusion of multidisciplinary, broad practitioner groups in each cohort of candidates, bringing fidelity to the interactions of the multidisciplinary team, as this would reflect what was experienced in the ‘real world’. This supports previous literature both in NLS and other areas of clinical practice, where multidisciplinary team training has been positively evaluated by practitioners, leading to enhanced teamwork and improved patient care (Capella et al., 2010; Hernández et al., 2021; Lavelle et al., 2018; Thomas et al., 2007, 2010; Weller et al., 2008). After eight months, several participants reported having used the training and described it as being ‘critical’ to the way they had managed the care episode. The course also appeared to have had an impact on practice that spread wider than the course attendees, through the uptake of best-practice NLS equipment in the participants’ organisations. Consistent with previous literature, the results suggested that availability of suitable equipment was a key barrier to confident management of UOHB (Goodwin et al., 2022; Persson et al., 2019). Candidates reported feeling empowered to explore ways to improve the availability of this equipment within their organisations. As a result of this OH-NLS course development, RCUK have produced a recommended minimum equipment list for UOHB (RCUK, 2023b). This has since been used by NHS England to update the recommended equipment list for UK ambulance services.

### Strengths and limitations

The evaluation was able to demonstrate the efficacy of a course specifically designed for out-of-hospital practitioners, and it is suggested that similar benefits could be achieved by delivering this type of course in other settings. The mixed-methods approach allowed a range of data to be collected to show how confidence was improved and to capture additional insights into the challenges faced by these professional groups attending UOHB.

Despite a high response rate, the number of participants on the course was relatively small and limited to two courses in one region of the UK, so there is limited external validity. The evaluation was not designed to report on objective competency but was focused on self-reported benefits to practice as perceived by participants. This has a risk of bias but was still of value as a means of determining educational benefit; however, further study to determine additional, more objective measures of sustained change in practice would be beneficial. There was also a risk of bias in the sample cohorts used, as they were the first volunteers of attendees on a new course, who may have been more motivated to learn and change their practice, as well as more aware of where their learning needs lie. Therefore, they may have performed better than later candidate cohorts and may have valued the course more highly, expressed through greater reported improvements in confidence. In addition, MCQ scores were not recorded pre-course to show improvement and were not repeated at the eight-month follow-up. While self-reported confidence remained high at the time point used, it is not known the full extent to which knowledge of NLS skills was sustained over time.

## Conclusion

The findings of this study suggest that the newly developed OH-NLS course improved participants’ knowledge and confidence in delivering care to stabilise a newborn baby outside the hospital setting. It is likely that it will contribute to raising the standards of care for babies born outside hospital, with the same success that the NLS course has for those born in hospital. Future research should explore the clinical impact of this training on outcomes for babies following UOHB, as well as long-term confidence and knowledge among practitioners.

## Acknowledgments

We would like to thank Health Innovation South West, New Life Special Care Babies and Resuscitation Council UK for supporting the development of this crucial course, and Health Innovation South West for supporting the evaluation. We would also like to thank all of the OH-NLS faculty and participants for their involvement in the proof-of-concept courses and this evaluation.

## Author contributions

MB, LH, SB, RT and LG were responsible for original design and conceptualisation; acquisition, analysis or interpretation of data; drafting, final approval and agreement to be accountable for all aspects of the work. DK, JM, LM, SH, WT, JY and AP were responsible for acquisition, analysis or interpretation of data, drafting, final approval and agreement to be accountable for all aspects of the work. MB acts as the guarantor for this article.

## Conflict of interest

The authors declare the following financial interests/personal relationships which may be considered as potential competing interests: RT and JM have unpaid roles on subcommittees for RCUK; JM has an unpaid role as Education Chair for the European Resuscitation Council NLS Science & Education Committee; MB has a paid role as Director of Clinical and Service Development at RCUK. The other authors declare that they have no known competing financial interests or personal relationships that could have appeared to influence the work reported in this article.

## Ethics

As this study involved healthcare professionals as participants, NHS REC Approval was not required. The HRA Decision Tool did not class this study as research, as it was an evaluation of a single course and findings were not intended to be generalisable. Therefore, HRA approval was not required. All participants provided informed consent for the use of their survey data within the evaluation.

## Funding

This evaluation was supported by Health Innovation South West as part of their Perinatal Equity Programme.
